# A novel STAT3/ NFκB p50 axis regulates stromal-KDM2A to promote M2 macrophage-mediated chemoresistance in breast cancer

**DOI:** 10.1186/s12935-023-03088-1

**Published:** 2023-10-11

**Authors:** Jia-Shing Chen, Yu-Ning Teng, Cheng-Yi Chen, Jing-Yi Chen

**Affiliations:** 1 School of Medicine for International Students, College of Medicine, I-Shou University, No.8, Yida Road, Jiaosu Village, Yanchao District, Kaohsiung, 82425 Taiwan; 2 School of Medicine, College of Medicine, I-Shou University, 8 Yida Road, Kaohsiung, 82445 Taiwan ROC; 3 Department of Pharmacy, E-Da Cancer Hospital, 21 Yida Road, Kaohsiung, 82445 Taiwan ROC; 4 Department of Cell Biology and Anatomy, College of Medicine, National Cheng Kung University, Tainan, 70101 Taiwan ROC; 5 Department of Medical Laboratory Science, College of Medical Science and Technology, I-Shou University, No.8, Yida Road, Jiaosu Village, Yanchao District, Kaohsiung, 82425 Taiwan ROC

**Keywords:** Lysine demethylase 2A, Cancer-associated fibroblasts, Tumor-associated macrophage, Paclitaxel resistance

## Abstract

**Background:**

Lysine Demethylase 2A (KDM2A) plays a crucial role in cancer cell growth, differentiation, metastasis, and the maintenance of cancer stemness. Our previous study found that cancer-secreted IL-6 can upregulate the expression of KDM2A to promote further the transition of cells into cancer-associated fibroblasts (CAFs). However, the molecular mechanism by which breast cancer-secreted IL-6 regulates the expression of KDM2A remains unclear. Therefore, this study aimed to elucidate the underlying molecular mechanism of IL-6 in regulating KDM2A expression in CAFs and KDM2A-mediated paclitaxel resistance in breast cancer.

**Methods:**

The ectopic vector expression and biochemical inhibitor were used to analyze the KDM2A expression regulated by HS-578 T conditioned medium or IL-6 in mammary fibroblasts. Immunoprecipitation and chromatin immunoprecipitation assays were conducted to examine the interaction between STAT3 and NFκB p50. M2 macrophage polarization was assessed by analyzing M2 macrophage-specific markers using flow cytometry and RT-PCR. ESTIMATE algorithm was used to analyze the tumor microenvironment-dominant breast cancer samples from the TCGA database. The correlation between stromal KDM2A and CD163 + M2 macrophages was analyzed using the Pearson correlation coefficient. Cell viability was determined using trypan blue exclusion assay.

**Results:**

IL-6 regulates gene expression via activation and dimerization of STAT3 or collaboration of STAT3 and NFκB. However, STAT3, a downstream transcription factor of the IL-6 signaling pathway, was directly complexed with NFκB p50, not NFκB p65, to upregulate the expression of KDM2A in CAFs. Enrichment analysis of immune cells/stromal cells using TCGA-breast cancer RNA-seq data unveiled a positive correlation between stromal KDM2A and the abundance of M2 macrophages. CXCR2-associated chemokines secreted by KDM2A-expressing CAFs stimulated M2 macrophage polarization, which in turn secreted CCL2 to increase paclitaxel resistance in breast cancer cells by activating CCR2 signaling.

**Conclusion:**

This study revealed the non-canonical molecular mechanism of IL-6 secreted by breast cancer upregulated KDM2A expression in CAFs via a novel STAT3/NFκB p50 axis, which STAT3 complexed with NFκB p50 in NFκB p50 binding motif of KDM2A promoter. KDM2A-expressing CAFs dominantly secreted the CXCR2-associated chemokines to promote M2 macrophage polarization and enhance paclitaxel resistance in breast cancer. These findings underscore the therapeutic potential of targeting the CXCR2 or CCR2 pathway as a novel strategy for paclitaxel-resistant breast cancer.

**Supplementary Information:**

The online version contains supplementary material available at 10.1186/s12935-023-03088-1.

## Background

Cancer-associated fibroblasts (CAFs), the most abundant components of the tumor microenvironment (TME), play critical roles in enabling tumors to evade the immune system, promoting the growth of tumor blood vessels, and facilitating metastasis. CAFs in the TME originate from various sources, including vascular endothelial cells, mesenchymal stem cells (MSCs), epithelial cells, and fibroblasts [1–3]. CAFs acquire their pro-tumorigenic potential through dysregulated epigenetic modifications. Normal fibroblasts can transform into CAFs when stimulated by paracrine signals such as cytokines, exosomes, and metabolites secreted by cancer cells. In contrast to normal-tissue-associated fibroblasts (NAFs), CAFs in prostate cancer exhibit elevated DNA hypermethylation of many tumor-associated genes [4]. Aberrant DNA methylation is associated with upregulation of CAFs-related genes, including fibronectin 1 and fibroblast activation protein (FAP) [5]. Hypomethylation of tumor suppressor genes, such as TGF-β1 receptor type 2 (TGFBR2) and PTEN, leads to suppressed expression of many genes in breast CAFs [6]. Additionally, aberrant expression of microRNAs (miRNAs) promotes the formation of CAFs in various tumors, such as ovarian, breast, lung, prostate, and gastric cancer [7–11]. Ectopic expression of miRNAs or using miRNA inhibitors may also induce the transformation of normal fibroblasts to CAFs. For example, dysregulated expression of the miR-200 family of miRNAs stimulates the expression of CAF-related genes, including α-smooth muscle actin (α-SMA) and FAP [7]. The expression of miR-31 and miR-214 was downregulated in ovarian CAFs, while the expression of miR-155 was upregulated [8]. Loss of EZH2 upregulates ADAMTS1 expression and promotes the transformation of normal fibroblasts into CAFs in breast cancer [12].

Lysine demethylase 2A (KDM2A), also known as FbxL11 due to its F-box and leucine-rich repeat domains, is responsible for demethylating histone H3 lysine 36 (H3K36) residues on unmethylated CpG islands. Studies have shown that KDM2A not only participates in the regulation of rRNA transcription and DNA repair [13, 14] but also plays a crucial role in promoting cell proliferation and maintaining heterochromatin status [15, 16]. Moreover, KDM2A is closely linked to the metastasis and progression of several cancers, including lung cancer, gastric cancer, cervical cancer, and glioblastoma [17–20]. Our previous study has shown that KDM2A promotes angiogenesis, maintains cancer stemness, and induces drug resistance in breast cancer by regulating jagged-1(JAG-1) and SRY-box transcription factor 2 (SOX-2). In breast cancer, KDM2A can promote DNA methylation by inhibiting the expression of tet-eleven translocation 2 (TET2), thereby suppressing the expression of epithelial cell adhesion molecule (EpCAM) and E-Cadherin, further enhancing the tumor invasion capacity of breast cancer [21, 22].

KDM2A is known to be upregulated in many cancers under the regulation of miRNAs. Studies have shown that miR-29b, miR-134-5p, and miR-3666 are recruited to the promoter region of KDM2A, leading to an increase in KDM2A expression [20, 23, 24]. In addition to miRNAs, factors related to inflammation, such as secreted frizzled-related protein 2 (SFRP2) and reactive oxygen species (ROS), can also upregulate the expression of KDM2A [25, 26]. Our recent study found that the pro-inflammatory cytokine IL-6, released by breast cancer cells, can increase the expression of KDM2A, thereby promoting the transformation of CAFs [27]. Furthermore, elevated KDM2A in CAFs leads to upregulation of PD-L1, which subsequently reduces the recruitment of natural killer (NK) cells to the TME [27]. However, the detailed molecular mechanism by which IL-6 regulates KDM2A in breast cancer remains unclear. Therefore, this study aimed to clarify the underlying molecular mechanism of IL-6-regulated KDM2A in enhancing chemoresistance in breast cancer. We demonstrated that KDM2A-expressing fibroblasts play an important role in cancer development and contribute to increased resistance to paclitaxel in breast cancer by promoting M2-macrophage polarization.

## Methods

### Cell culture and reagents

Immortalized breast fibroblasts RMF-EG cell line, dominant-negative STAT3 (DN-STAT3) plasmids, and constitutively active STAT3 (pSTAT3) plasmids were kindly provided by Dr. Wen-Chun Hung (National Institute of Cancer Research, National Health Research Institutes, Taiwan). THP-1, MDA-MB-231, and HS-578 T cell lines were purchased from the Bioresource Collection and Research Center (BCRC). KDM2A overexpression in RMF-EG fibroblasts (K2A-1) was established as previously described [27]. RMF-EG, MDA-MB-231, and HS-578 T cells were cultured with DMEM medium supplemented with 10% fetal bovine serum (FBS). THP-1 was cultured with RPMI-1640 medium supplemented with 10% FBS and 2 mM L-glutamine. IL-6, CXCL2, CXCL5, and IL-8 were purchased from Cell Guidance Systems, LLC. (St. Louis, MO, USA). Chromatin immunoprecipitation (ChIP) kit (catalog #ab500) and anti-KDM2A antibody were obtained from Abcam (Cambridge, MA, USA). Antibodies against STAT3, phospho-STAT3 (Tyr 705), and NFκB p50 were purchased from Cell Signaling Technology Inc. (Danvers, MA, USA). Anti-actin antibody was obtained from Novus Biologicals, LLC. (Centennial, CO, USA). Alexa Fluor 488 anti-CD206 and Alexa Fluor 647 anti-CD163 antibodies were purchased from BioLegend, Inc. (San Diego, CA, USA). Stattic, paclitaxel, SB225005, and INCB3344 were purchased from MedChemExpress, LLC. (Monmouth Junction, NJ, USA). NF-κB inhibitor JSH-23 was obtained from Cayman Chemical Company (Ann Arbor, Michigan, USA). ViaFect transfection reagent was purchased from Promega Corporation (Madison, WI, USA).

### Preparation of conditioned medium

HS-578 T (7 × 10^6^ cells), RMF-EG (5 × 10^6^ cells), and K2A-1 cells (5 × 10^6^ cells) were seeded in 10 cm-dish. After reaching about 70% confluency, cells were washed using PBS and then cultured in a serum-free medium for 48 h. The medium was then collected, centrifuged to remove remnant cells and debris, and stored at –80 °C as conditioned medium (named HS-578 T-CM, RMF-CM, and K2A-CM in this study). PMA-pretreated THP-1 cells were co-cultured with K2A-1 cells using a transwell chamber. THP-1 cells (1 × 10^6^ cells) were grown in the lower chamber, while K2A-1 cells (1 × 10^5^ cells) were seeded on the polyester membrane (pore size: 1 µm) of the transwell insert. The cells in the transwell chamber were incubated in a humidified incubator at 37 °C and 5% CO2. After 72 h of co-culture, THP-1 cells were washed with PBS and then cultured in a serum-free RPMI-1640 medium. After an additional 48 h, the supernatants of THP-1 cells and K2A-1 cells were collected as conditioned medium and named TRMF-CM and TRK2A-CM, respectively.

### Co-culture of THP-1 with K2A-1

THP-1 cells were treated with 50 ng/ml phorbol-12-myristate-13-acetate (PMA) for 24 h and then co-cultured with K2A-1 cells in a transwell chamber. THP-1 cells (1 × 10^6^) were grown in the lower chamber, while K2A-1 cells (1 × 10^5^) were seeded on the polyester membrane (pore size: 1 µm) in the transwell insert. The cells in the transwell chamber were incubated in a humidified incubator at 37 °C and 5% CO2. After an additional 72 h of co-culture, the total RNAs of the THP-1 cells were extracted, and M1- and M2-macrophage-related markers were determined by quantitative reverse transcription-polymerase chain reaction (qRT-PCR).

### Transfection

The DN-STAT3 or pSTAT3 plasmids (1 μg) were transfected into RMF-EG cells using ViaFect transfection reagent (Promega Corporation, Madison, WI, USA). After 24 h of transfection, the cells were further incubated with 20 ng/ml IL-6 or HS-578 T-CM for 24 h, and stattic (5 μM) or JSH-23 (30 μM) was added simultaneously according to the experimental design. Afterward, total proteins and total RNAs of the cells were extracted and subjected to Western blot analysis and qRT-PCR assay.

RMF-EG cells were transfected with pSTAT3 plasmids for 24 h and then incubated with 30 μM JSH-23 for an additional 24 h. Afterward, total proteins and total RNAs of the cells were extracted and subjected to Western blot analysis and qRT-PCR assay.

### RNA extraction and qRT-PCR

Total RNAs were extracted using an RNA extraction kit (Geneaid Biotech Ltd). Briefly, 1 μg of extracted RNAs was reversely transcribed into complementary DNA (cDNA) by M-MLV reverse transcriptase. The relative expression of target genes was determined and quantified by qRT-PCR (SYBR Green approach). The expression of β-actin was used as an internal control for normalization. The PCR cycling condition for detecting target genes included an initial denaturation at 95 °C for 5 min, followed by 30 cycles of 95 °C for 45 s, 60 °C for 45 s, and 72 °C for 45 s, and a final cycle of 72 °C for 10 min. Primer sets used to detect genes of interest are listed in the Additional file 5: Table S1.

### ChIP assay

ChIP assay was performed using the ChIP kit (Abcam, ab500). IL-6-treated RMF-EG cells were lysed, and genomic DNAs were sheared by sonication. Fragmented DNA samples were divided into a test group and a negative control group. The remaining DNA samples were used as the input group. DNA samples were added with anti-STAT3 or non-immune (negative control) antibodies and incubated overnight at 4 °C with rotation. Antibody-bound protein/DNA complexes were pulled down by protein A/G beads. The pulled-down DNA fragments were recovered and subjected to PCR to detect the presence of STAT4 and NFκB p50 binding sites on the KDM2A promoter region. Primer sets specific for STAT4 and NFκB p50 binding sites were listed in the Additional file 5: Table S1.

### Immunoprecipitation and Western blotting analysis

RMF-EG cells were treated with or without IL-6 (20 ng/ml) for 48 h and then lysed with RIPA buffer (50 mM Tris–HCl, pH 7.4, 150 mM NaCl, 1% NP-40, 0.1% SDS, 0.5% sodium deoxycholate, 2 mM EDTA, and 50 mM NaF) containing protease inhibitors. After determining the protein concentration of the lysate by bicinchoninic acid (BCA) assay, 1 mg protein was incubated with anti-STAT3 antibody overnight at 4 °C with rotation. Antibody-protein complexes were pulled down by protein A/G beads, washed three times with RIPA buffer, and eluted with sample buffer for 10 min in a dry block heater. The eluted protein samples were resolved by SDS-PAGE electrophoresis and transferred to PVDF membranes. The blots were immunoblotted with anti-NFκB1p50 or anti-STAT3 antibodies. The blots were washed and then developed by enhanced chemiluminescence reagent.

For Western blotting analysis, total proteins were extracted using RIPA lysis buffer containing protease inhibitors. After determining the protein concentration using BCA assay, 20 μg of total protein was resolved by SDS-PAGE, transferred to PVDF membranes, immunoblotted with indicated primary antibodies and corresponding secondary antibodies, and developed by enhanced chemiluminescence reagent. Actin was used as an internal control for normalization.

### Assessment of stromal and immune cell infiltration and gene expression profile

The RNA-seq data of 1026 breast cancer samples were obtained from the TCGA database. Transcripts per kilobase million (TPM) was used as the measure for transcriptional levels. The degree of stromal cells and infiltrating immune cells in the TME of breast cancer and breast tumor purity were calculated by StromalScore, ImmuneScore, and ESTIMATEScore. Breast tumor samples with an ESTIMATEScore and ImmuneScore to StromalScore ratio (I:S ratio) above the median were classified as the immune/stromal-rich group and used to calculate the correlation between KDM2A and CD163.

### Cytotoxicity assay

HS-578 T cells or MDA-MB-231 cells (10,000 cells) were incubated with THP-CM, TRMF-CM, or TRK2A-CM and treated with paclitaxel with or without 5 nM INCB3344 for 48 h. The dosage of paclitaxel triggered cell apoptosis was referred to a previous study [49]. Cells were harvested, and viable cells were determined and counted using the trypan blue exclusion assay.

### CD163/CD206 M2 macrophage population assay with flow cytometry assay

Co-cultured THP-1/RMF-EG cells and THP-1/K2A-1 cells were fixed with 3.7% formaldehyde for 15 min at room temperature. After washing three times with 1 × PBS, fixed cells were incubated with anti-CD163 antibody conjugated with Alexa Fluor 488 and anti-CD206 antibody conjugated with Alexa Fluor 647 for 1 h at room temperature. After washing away the unbound antibodies, the number of CD163/CD206 double-positive cells was detected and quantified by flow cytometry (Attune™ NxT Flow Cytometer).

### Statistical analysis

Statistical comparisons were performed using Student’s t-test to investigate differences between groups. Data are expressed as the mean ± standard error of the mean (SEM). The correlation between KDM2A and CD163 was examined by calculating Pearson's correlation coefficient. A *p*-value < 0.05 was considered statistically significant. All statistical analyses in this study were performed using GraphPad Prism version 5.01 (GraphPad Software, Inc.).

## Results

### IL-6 induces the expression of KDM2A and promotes the transformation of normal mammary fibroblasts into CAFs via STAT3

**Fig. 1:**
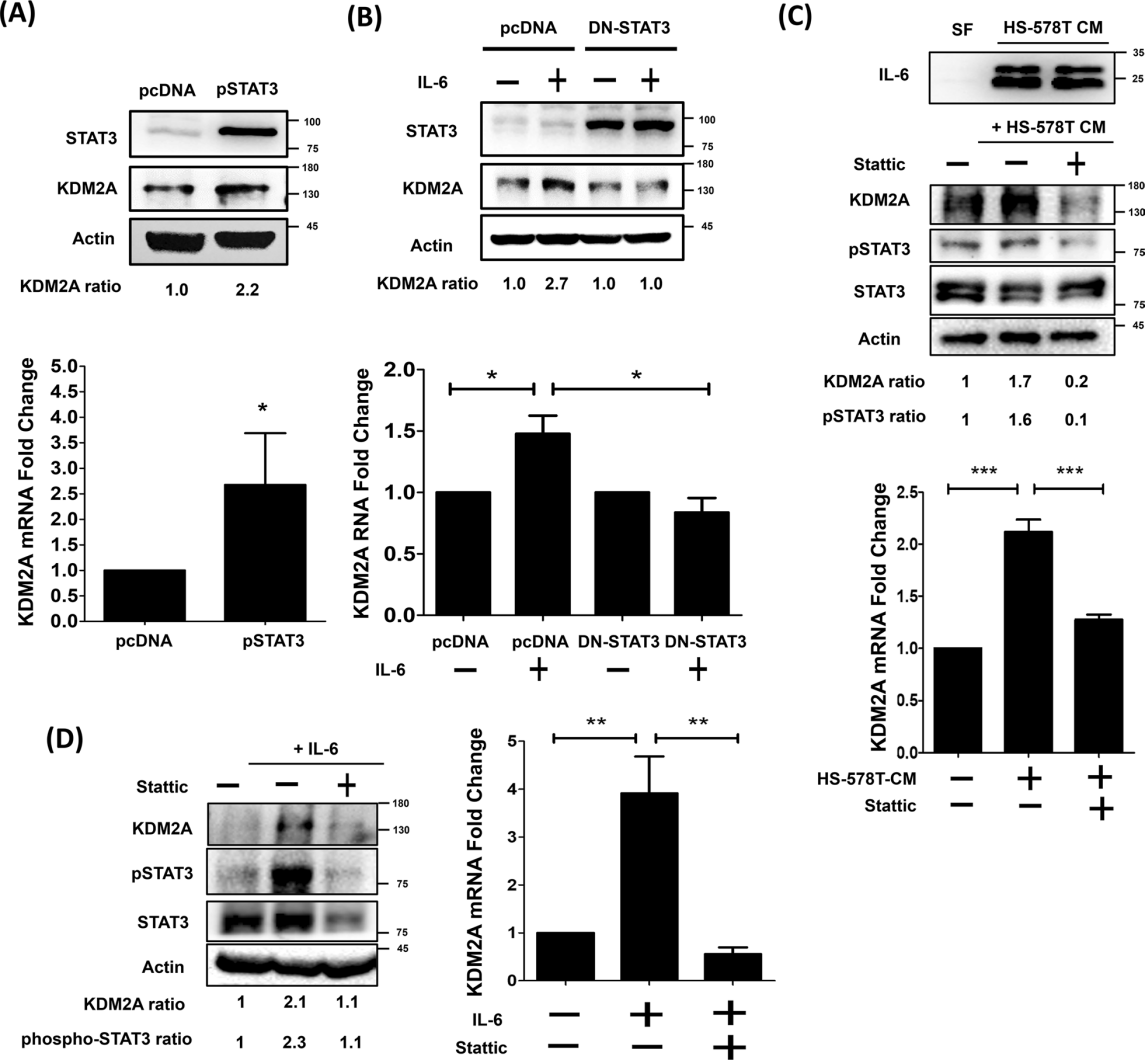
IL-6 upregulated the expression of KDM2A in mammary fibroblasts by activating STAT3. **A** Overexpression of constitutively active STAT3 by pSTAT3 plasmid increased the expression of KDM2A in RMF-EG cells. The protein and mRNA levels of KDM2A were analyzed by Western blotting analysis and real-time PCR, respectively. **B** RMF-EG cells were transfected with dominant-negative STAT3 plasmids and then treated with or without 10 ng/ml IL-6 for 48 h. The protein and mRNA levels of KDM2A were analyzed by Western blotting analysis and real-time PCR, respectively. The pcDNA3.1 vector was used as the control vector. **C** RMF-EG cells were incubated with HS-578 T CM for 48 h in the presence or absence of 5 μM stattic. The protein and mRNA levels of KDM2A were analyzed by Western blotting analysis and real-time PCR, respectively. IL-6 expression in serum-free medium (SF) or HS-578 T CM was analyzed by Western blotting analysis. **D** RMF-EG cell was treated with 20 ng/ml IL-6 for 48 h in the presence or absence of 5 μM stattic. The protein and mRNA levels of KDM2A expression were analyzed by Western blotting analysis and real-time PCR, respectively. Differences were found to be statistically significant at * p < 0.05, ** p < 0.01, and *** p < 0.001

In our previous study, the transcriptional level of KDM2A was increased in normal fibroblasts after stimulation with tumor-derived inflammatory cytokines TNF-α and IL-6 and further drove the transformation of the cells into CAFs [27]. However, the underlying molecular mechanism remains unclear. Therefore, this study aimed to explore the molecular mechanism by which IL-6 regulates the expression of KDM2A in CAFs. To understand whether IL-6 increases KDM2A expression by STAT3 signaling, pSTAT3 or DN-STAT3 plasmids were transfected into mammary fibroblast RMF-EG cells. As shown in Fig. 1A, ectopic expression of pSTAT3 significantly increased KDM2A mRNA levels by about 2.5-fold (P < 0.05). In addition, KDM2A protein levels were also raised by approximately 2.2-fold. On the other hand, IL-6 treatment increased KDM2A expression, while ectopic expression of DN-STAT3 further suppressed IL-6-induced KDM2A expression (Fig. 1B). Afterward, STAT3 inhibitor stattic was used to inhibit the endogenous IL-6/STAT3 pathway in HS-578 T CM-treated RMF-EG cells (Fig. 1C) and IL-6-stimulated RMF-EG cells (Fig. 1D). After blocking STAT3 activation by stattic, the elevated KDM2A expression induced by IL-6 and HS-578 T CM treatment was dramatically reduced, suggesting that IL-6 can activate STAT3 and transcriptionally upregulate the expression of KDM2A in CAFs.

### IL-6 regulates KDM2A expression by activating NFκB1 in mammary fibroblasts

**Fig. 2:**
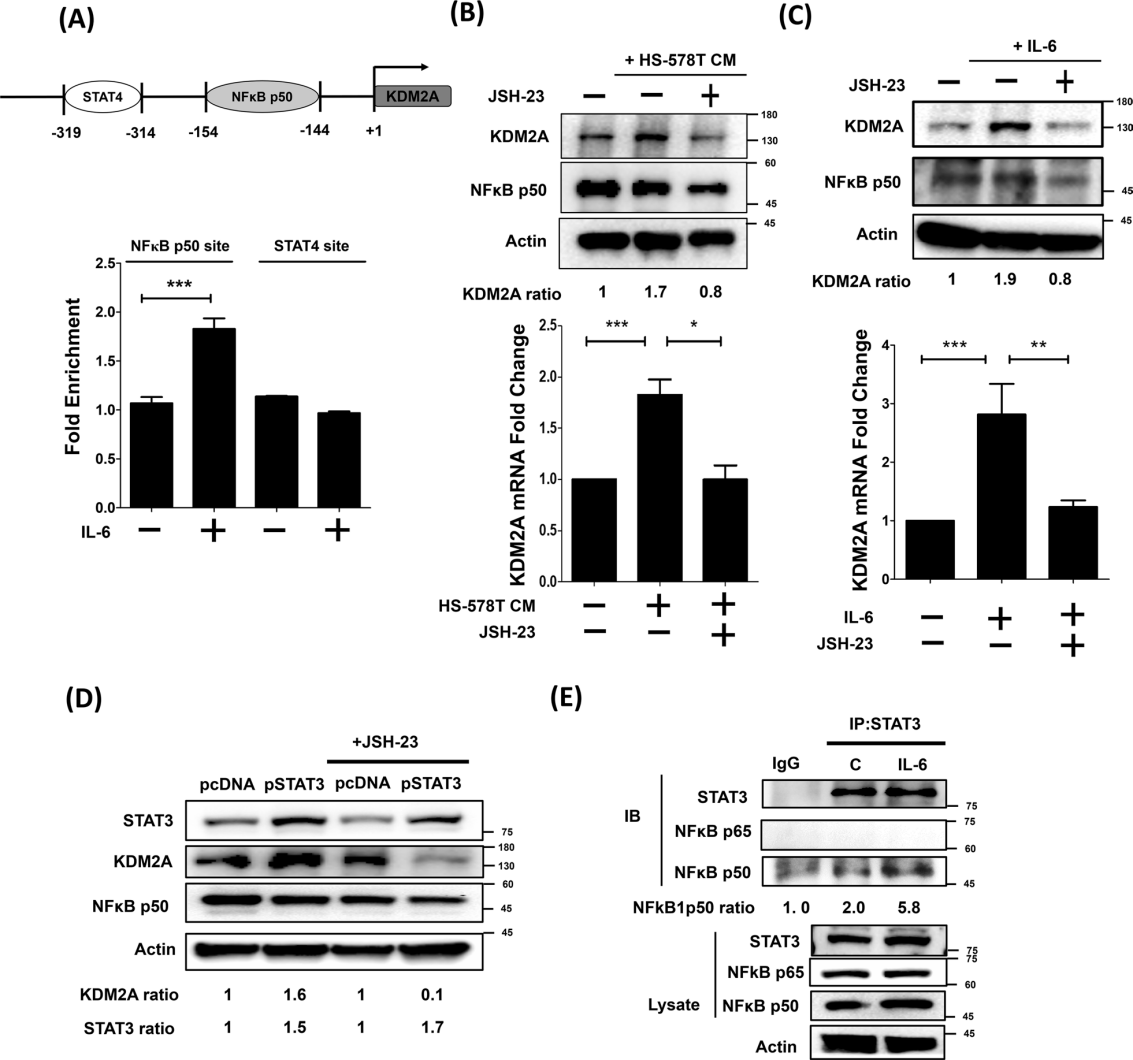
IL-6 upregulated the expression of KDM2A in mammary fibroblasts through the STAT3/NFκB p50 axis. **A** ChIP assay was performed to pull down the STAT3 proteins-chromatin complexes in IL-6-treated mammary fibroblasts using anti-STAT3 antibody. STAT4 and NFκB p50 binding motifs on the KDM2A promoter region were amplified by PCR. Each experiment was performed in triplicate and repeated three times independently. Data are expressed as the fold change relative to non-treated control cells. The upper region of the histogram shows the putative STAT4 and NFκB p50 binding motif on the KDM2A promoter region. **B** RMF-EG cells were incubated with HS-578 T CM for 48 h in the presence and absence of JSH-23. The protein and mRNA levels of KDM2A were analyzed by Western blotting analysis and real-time PCR, respectively. **C** RMF-EG cells were treated IL-6 for 48 h in the presence and absence of 30 μM JSH-23. The protein and mRNA levels of KDM2A were analyzed by Western blotting analysis and real-time PCR, respectively. **D** Ectopic pSTAT3-expressing RMF-EG cells were treated with or without JSH-23 for 48 h. KDM2A protein level was analyzed by Western blotting analysis. **E** Immunoprecipitation assay was performed in IL-6-treated RMF-EG cells. STAT3 bound protein complexes were pulled down using anti-STAT3 antibody and analyzed by immunoblotting with anti-NFκB p65 and anti-NFκB p50 antibodies. Differences were found to be statistically significant at *p < 0.05, ** p < 0.01, and *** p < 0.001

To understand whether STAT3, the downstream molecule of the IL-6 signaling pathway, regulates the expression of KDM2A by directly binding to the KDM2A promoter region, the STAT3 binding site on the KDM2A promoter region was predicted using Promo3.0 online software. No STAT3 binding site was predicted on the promoter region of KDM2A. However, one STAT4 and one NFκB p50 binding site were predicted on the − 319 bp to − 314 bp region and − 154 bp to − 144 bp region of the KDM2A promoter, respectively. Next, ChIP assay was used to confirm whether STAT3 upregulates the expression of KDM2A in HS-578 T CM- or IL-6-treated RMF-EG cells by binding to STAT4 or NFκB p50 binding sites. Results of STAT3-pull-down experiments showed that the NFκB1 binding sites that could bind to STAT3 were significantly enriched in IL-6-treated RMF-EG cells (P < 0.001; Fig. 2A, lower panel). However, there was no significant difference in the enrichment of STAT4 binding sites between cells with and without IL-6 treatment. Similar results were observed in HS-578 T CM-treated RMF-EG cells (Additional file 1: Figure S1). Next, JSH-23 inhibitor was used to inhibit the nuclear translocation of NFκB to further verify whether NFκB p50 was involved in the expression of KDM2A regulated by STAT3. As shown in Fig. 2B, blocking NFκB nuclear translocation by JSH-23 effectively suppressed elevated mRNA and protein levels induced by HS-578 T CM. Similar results were observed in IL-6-treated RMF-EG cells (Fig. 2C). Furthermore, inhibition of NFκB nuclear translocation by JSH-23 significantly abolished the upregulation of KDM2A in ectopic STAT3-expressed RMF-EG cells (Fig. 2D). Next, co-immunoprecipitation assays were used to clarify whether STAT3 directly interacts with NFκB p50 in IL-6-treated RMF-EG cells. As shown in Fig. 2E, increased co-immunoprecipitated NFκB p50 was observed in IL-6-treated RMF-EG cells. Collectively, the above results suggest that IL-6 regulates the expression of KDM2A in CAFs through the STAT3/NFκB p50 axis.

### Stromal KDM2A expression was positively correlated with the enrichment of infiltrated CD163^+^ M2 macrophages in TME

**Fig. 3:**
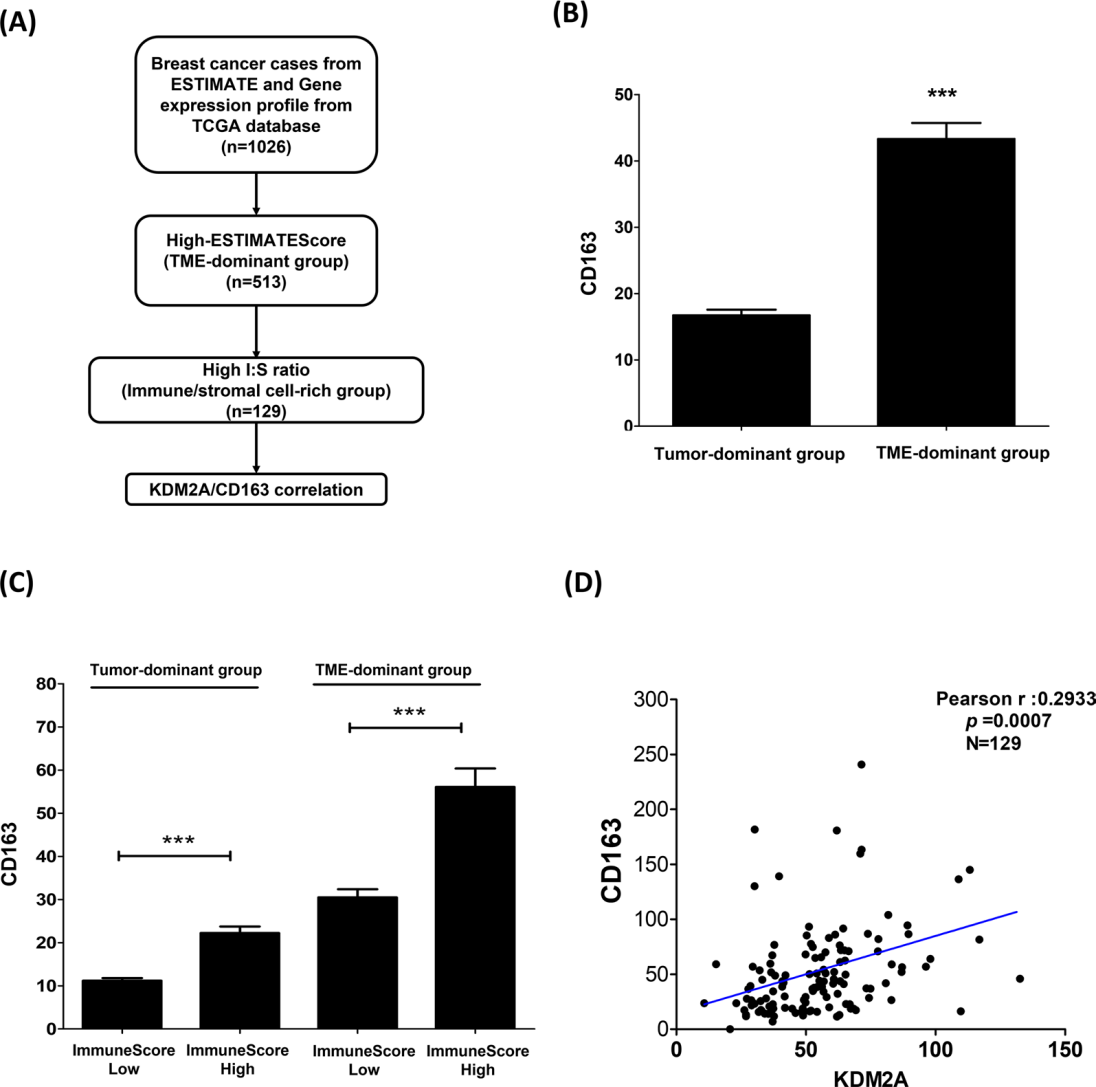
Stromal KDM2A was associated with infiltrated CD163 + M2 macrophages in the breast tumor microenvironment. **A** Flowchart of analyzing gene expression profiles of breast tumor samples obtained from the TCGA database. The abundance of stromal and infiltrated immune cells in each breast tumor sample was assessed using ESTIMATEScore, StromalScore, and ImmuneScore. **B** According to ESTIMATEScore, the samples were divided into tumor-dominant group and TME-dominant group, and the relative expression levels of CD163 in the two groups were analyzed. **C** The abundance of CD163 + cells was analyzed between the two groups and further stratified by ImmuneScore. **D** The correlation between the stromal KDM2A and CD163^+^ M2 macrophage was evaluated by the Person’s correlation coefficient in the stromal and immune cell-high group. The I:S ratio was calculated as the ratio of ImmuneScore to StromalScore and used to represent immune and stromal content in the TME-dominant group. Differences were found to be statistically significant at * p < 0.05, ** p < 0.01, and *** p < 0.001

Previous studies demonstrate that cytokines secreted by CAFs can activate macrophages and promote M2 macrophage polarization, thereby promoting tumor progression [32–35]. To understand whether KDM2A-expressing fibroblasts promote M2 macrophage polarization, we analyzed gene expression profiles obtained from the TCGA database. Figure 3A shows the flow diagram for assessing the abundance of stromal and infiltrated immune cells in the TME in each breast tumor sample using StromalScore, ImmuneScore, and ESTIMATEScore. The median ESTIMATEScore of our study tumor samples was 495.26, which was used as a cut-off value to divide breast tumor samples into the TME-dominant group (≥ 495.26) and tumor-dominant group (< 495.26). CD163, a marker of M2 macrophages, was used to analyze the enrichment of M2 macrophages in the two groups. As shown in Fig. 3B, the TME-dominant group showed a significantly higher abundance of M2 macrophages compared to the tumor-dominant group (P < 0.001). Subsequently, the abundance of CD163^+^ M2 macrophages in the two groups was stratified by ImmuneScore and StromalScore. As shown in Fig. 3C, regardless of the tumor-dominant group or the TME-dominant group, the abundance of CD163^+^ M2 macrophages was significantly enriched in the high ImmuneScore subgroup compared to the low ImmuneScore subgroup (P < 0.001). However, there was no significant difference in the abundance of CD163^+^ M2 macrophages between the high and low StromalScore subgroups (P > 0.05, Additional file 2: Figure S2). These results indicated that in the TME-dominant group with enriched CD163^+^ M2 macrophages, these M2 macrophages were mainly distributed in the high ImmuneScore subgroup rather than the high StromalScore subgroup. Therefore, 256 samples with high ImmuneScore in the TME-dominant group were selected for further analysis. To analyze the correlation between stromal-KDM2A and CD163^+^ M2 macrophages, the ratio of ImmuneScore to StromalScore (I:S ratio) was calculated to assess the content of stromal and immune cells in the high-stromal group. The median I:S ratio was 1.384473 and was further used as a cutoff value to divide the samples into the low and high I:S ratio groups. Since a high I:S ratio indicates the presence of both immune and stromal cells, there were 129 samples in the TME-dominant group that had a high I:S ratio and were referred to as the immune/stromal cell-rich group. Afterward, the Pearson correlation coefficients of KDM2A and CD163 were evaluated in the immune/stromal cell-rich group, and the results showed a positive correlation between KDM2A and CD163 (Pearson r = 0.29, P < 0.001, Fig. 3D). In summary, stromal-KDM2A was positively associated with enriched CD163^+^ M2 macrophages in the TME.

### KDM2A-expressing fibroblasts drive M2 macrophage polarization via CXCR2 signaling and promote paclitaxel resistance in breast cancers through CCL2/CCR2 signaling

**Fig. 4:**
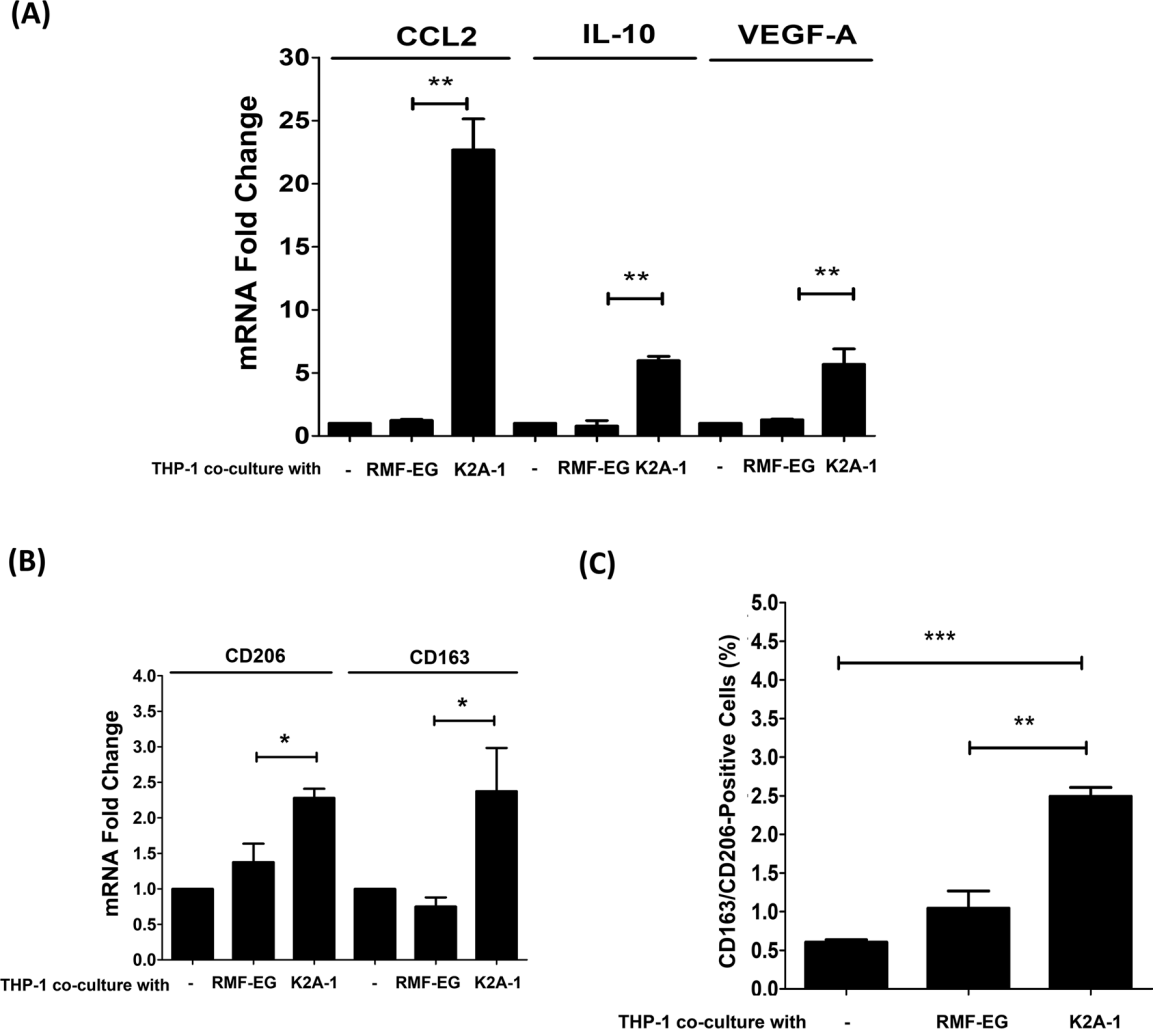
KDM2A-expressing mammary fibroblasts promoted M2 macrophage polarization. **A** PMA-pretreated THP-1 cells were co-cultured with RMF-EG cells or KDM2A-expressing RMF-EG cells (K2A-1), and the relative expression of M2-macrophage markers CCL2, IL-10, and VEGF-A were determined by real-time PCR. **B** Relative mRNA levels of tumor-associated fibroblast surface marker CD206 and CD163 in THP-1 cells co-cultured with RMF-EG or KDM2A-expressing RMF-EG (K2A-1) cells were determined by real-time PCR. **C** The relative expression levels of CD206 and CD163 on the cell surface of THP-1 cells co-cultured with RMF-EG or KDM2A-expressing RMF-EG (K2A-1) cells were determined and quantified by flow cytometry. Each experiment was performed in triplicate and repeated three times independently. Differences were found to be statistically significant at * p < 0.05, ** p < 0.01, and *** p < 0.001

To explore whether KDM2A-expressing fibroblasts could promote the differentiation of tumor-associated macrophages, unique markers associated with M1 and M2 macrophages were analyzed in THP-1 cells co-cultured with K2A-1 cells. As shown in Fig. 4A, the expression levels of M2 macrophage markers CCL2, IL-10, and VEGF-A in THP-1 cells were significantly increased when co-cultured with K2A-1 cells (All P < 0.01). In contrast, the expression of nitric oxide synthase (NOS-2), a marker of M1 macrophages, was significantly decreased (P < 0.05, Additional file 3: Figure S3). The results were further confirmed by examining two surface markers of M2 macrophages, CD163 and CD206, using real-time PCR and flow cytometry. Consistently, K2A-1 co-culture significantly increased the mRNA (P < 0.05, Fig. 4B) and surface expression (P < 0.01, Fig. 4C) of CD163 and CD206 in THP-1 cells. Collectively, the results suggest that KDM2A-expressing fibroblasts can promote M2 macrophage polarization.

**Fig. 5:**
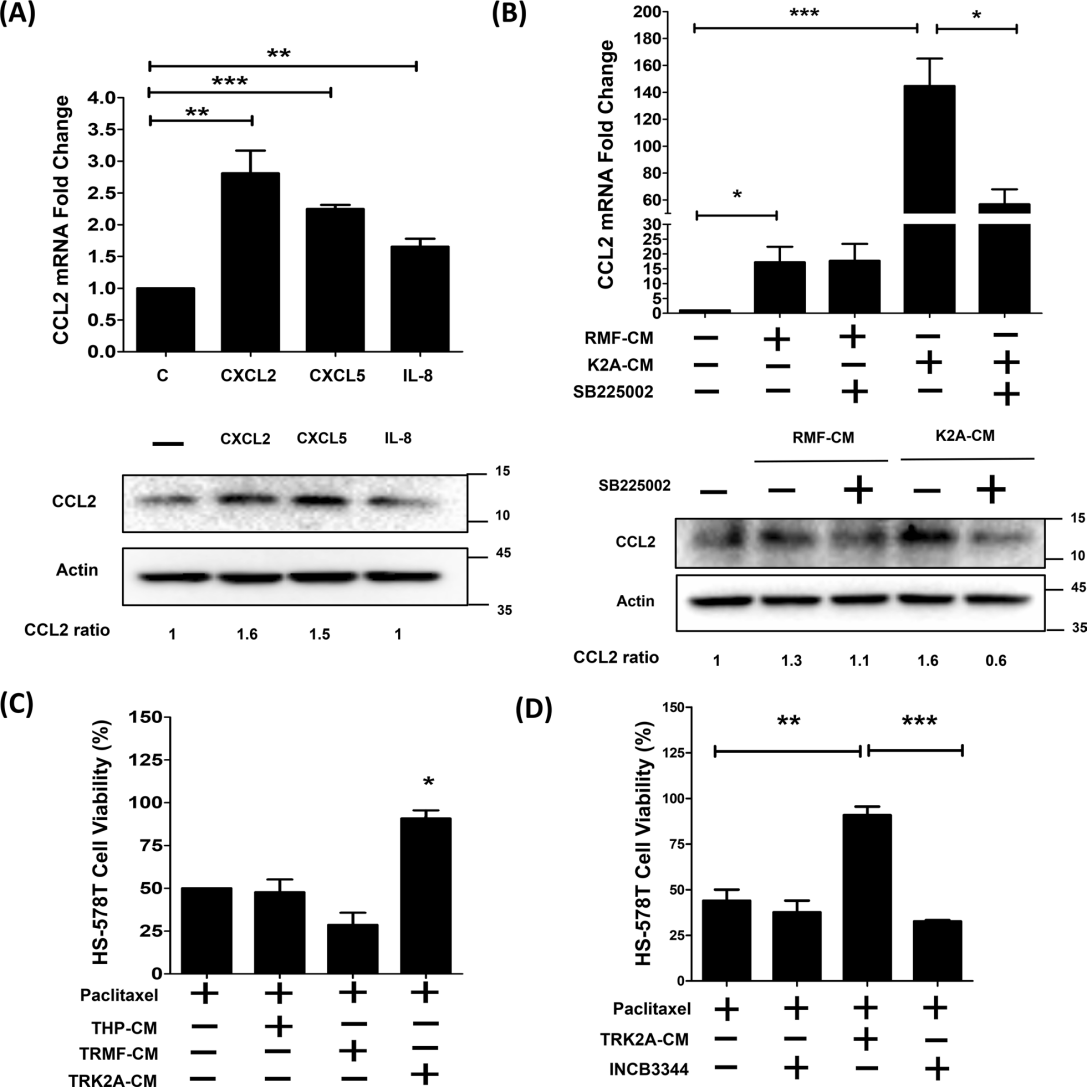
KDM2A-expressed fibroblasts stimulated CCL2 released from macrophage through CXCR2 to increase the paclitaxel resistance in breast cancer. **A** PMA-pretreated THP-1 was treated with 100 ng/ml CXCL2, CXCL5, or IL-8, and relative mRNA and protein levels of CCL2 were determined by Real-time PCR and western blot analysis. **B** PMA-pretreated THP-1 cells were incubated with RMF-CM or K2A-CM in the presence and absence of CXCR2 inhibitor SB225002 (25 nM). Relative mRNA and protein levels of CCL2 were analyzed by Real-time PCR and western blot analysis. **C** Cell viability analysis of paclitaxel-treated HS-578 T cells. HS-578 T cells were treated with conditioned medium TRMF-CM or TRK2A-CM supplemented with additional 25 nM paclitaxel for 48 h. **D** Cell viability of paclitaxel-treated HS-578 T cells co-treated with TRK2A-CM in the presence and absence of CCR2 inhibitor INCB3344 (5 nM) for 48 h. Cell viability was determined and counted using the trypan blue exclusion assay. Each experiment was performed in triplicate and was repeated three times independently. Differences were found to be statistically significant at * p < 0.05, **p < 0.01, and ***p < 0.001

The mechanism by which KDM2A promotes the polarization of M2 macrophages may be attributed to paracrine factors secreted by co-cultured K2A-1 cells. In our previous microarray study of the expression profile of K2A-1 cells, the expression levels of CXCR2-associated chemokines, such as CXCL2, CXCL5, and IL-8, were increased in K2A-1 cells [27]. To understand whether CXCL2, CXCL5, and IL-8 cytokines secreted by K2A-1 cells can stimulate M2 macrophage polarization, THP-1 cells were treated with recombinant CXCL2, CXCL5, and IL-8. As shown in Fig. 5A, the transcriptional levels of the M2 macrophage marker CCL2 were significantly increased after treatment with CXCL2, CXCL5, or IL-8 (P < 0.05). The CCL2 protein was 1.6- and 1.5-fold increase in the CXCL2- and CXCL5-treated THP-1 cells. Next, we examined whether the conditional medium of K2A-1 (K2A-CM) could also upregulate the expression of CCL2 in THP-1 cells and further explored whether this increase was achieved through the CXCR2 pathway. As shown in Fig. 5B, K2A-CM treatment significantly increased the expression of CCL2 (P < 0.001). However, inhibition of CXCR2 signaling by SB225002 significantly suppressed K2A-CM-induced CCL2 expression (P < 0.05).

Since CCL2 secreted by M2 macrophages is known to be associated with paclitaxel resistance in triple-negative breast cancer (TNBC), we next examined whether CCL2 secreted by THP-1 cells co-cultured with K2A-1 is involved in the paclitaxel resistance mechanism. Cell viability of HS-578 T and MDA-MB-231 TNBC cells was measured after co-treatment with paclitaxel plus TRMF-CM or TRK2A-CM. As shown in Fig. 5C, TRK2A-CM significantly restored the cell viability of paclitaxel-inhibited HS-578 T TNBC cells (P < 0.05). A similar observation was observed in MDA-MB-231 TNBC cells (P < 0.05, Additional file 4: Figure S4). Next, the CCR2 inhibitor INCB3344 was used to confirm that the effect of TRK2A-CM in restoring paclitaxel-inhibited cell viability was through CCL2 signaling. As shown in Fig. 5D, restored cell viability by TRK2A-CM was significantly suppressed after the addition of INCB3344. Collectively, these results suggest that CXCR2-associated chemokines released from K2A-1 promote M2 macrophage polarization, which in turn secretes CCL2 to increase the resistance of breast cancer cells to paclitaxel.

## Discussion

Inflammatory tumor microenvironment is conductive to tumor progression. This study demonstrates that the proinflammatory cytokine IL-6 upregulates the epigenetic regulator KDM2A through a non-canonical pathway, the STAT3/NFκB p50 axis in mammary fibroblasts. KDM2-expressing CAFs mediate breast cancer paclitaxel resistance by driving M2-like macrophage polarization. Tumor-derived factors can activate NAFs and transform them into tumorigenic CAFs. For example, prostate carcinoma-secreted IL-6 can increase the expression of the cancer-associated fibroblast marker FAP, activating human prostate fibroblasts and enhancing the invasive ability of prostate cancer [28]. Breast and pancreatic cancer cells can transform normal dermal fibroblasts into CAFs through the IL-1β/NFκB signaling cascade, thereby enhancing the inflammatory TME [29]. Despite these studies, the molecular mechanisms by which tumor-secreted factors regulate the transformation of NAFs into CAFs remain poorly understood. A study by Albrengues et al. found that the pro-inflammatory leukemia inhibitory factor (LIF) can promote the transcription of DNMT and recruit p300 histone acetyltransferases (HAT) to trigger the constitutive activation of JAK1/STAT3 signaling, thereby maintaining the phenotype and function of CAFs [5]. Our previous study also showed that breast cancer-secreted TNF-α and IL-6 can drive the transformation of mammary fibroblast cells into CAFs by upregulating the expression of KDM2A and subsequently upregulating the expression of PD-L1, providing an immunosuppressive TME for breast cancer progression [27].

The inflammatory tumor microenvironment is beneficial for tumor progression. The Zhe Ji groups have demonstrated that the inflammatory regulation network mediated by NFκB, STAT3, and AP-1 factors is essential in many cancers. They have suggested that NFκB p50 complexes with NFκB p65, STAT3, and AP-1 factors in the nucleus of transformed mammary cells. However, NFκB p50, STAT3, and AP-1 have not been colocalized in the same *cis*-regulatory regions [30]. IL-6, as a proinflammatory cytokine, plays a pivotal role in tumor progression, including breast, lung, ovarian, colorectal, and pancreatic cancer. In the canonical pathway, IL-6 can activate the JAK2/STAT3 signaling cascade to regulate a series of downstream genes, such as NFκB, which collectively control tumorigenesis. Some studies have shown that STAT3 can interact with the NFκB p65 subunit and bind to the promoter regions of various genes. Radiation can further increase the interaction of the nuclear STAT3/ NFκB p65 complex with the proximal intron-1 region of the ICAM-1 gene in glioblastoma multiforme [31]. In addition, NFκB p65 regulated by IL-1 can cooperate with STAT3 to bind to distal cis-regulatory elements of IL-17, thereby promoting IL-17 transcription and Th17 differentiation [32]. In addition to STAT3 interacting with NFκB p65, the Chan-Wei Yu groups have demonstrated that IL-6 activated STAT3/NFκB p50 signaling to recruit HDAC1 to the circadian gene Period2 promoter, suppressing the expression of Period2 in mammary epithelial cells. They have indicated that activated-STAT3 promotes the nucleus translocation and chromatin binding of NFκB p50 [33]. We showed that IL-6 regulated the expression of KDM2A in CAFs through the STAT3/NFκB pathway. However, only the NFκB p50 binding site, not the NFκB p65 or STAT3 binding site, was found in the KDM2A promoter region. We hypothesized that activated-STAT3 interacted with NFκB p50 in the NFκB p50 binding site of the KDM2A promoter region. Our pull-down experiments further verified that STAT3 interacted with NFκB p50 but not NFκB p65 to bind the promoter region of KDM2A. Therefore, the results of this study demonstrate a novel collaboration of STAT3 and NFκB p50 in promoting the regulation of KDM2A in CAFs.

The presence of CAFs promotes the development of an inflammatory TME conductive to cancer development. Tumor-associated macrophages (TAMs), differentiated from monocytes or resident macrophages, play a crucial role in tumor-associated inflammation when infiltrating the TME. M2-like TAMs contribute to tumorigenesis by secreting soluble factors such as VEGF, IL-10, and CCL2. In the context of oral squamous cell carcinoma (OSCC), CAFs-released IL-6 and GM-CSF drive the activation of M2-like TAMs in the TME [34]. In a study of pancreatic ductal adenocarcinoma by Zhang et al., CAFs-secreted M-CSF increased ROS production and promoted M2 macrophage polarization, thereby facilitating pancreatic cancer growth [35]. In hepatocellular cancer, CAFs-secreted CXCL12 drives the M2 macrophage polarization and the release of plasminogen activator inhibitor‑1 (PAI-1), ultimately promoting tumor progression [36]. Moreover, CAFs-secreted CXCL12 recruits monocytes and induces their differentiation into M2-like macrophages, a crucial factor in enhancing cancer stemness and resistance to chemotherapy [37]. In this study, we further elucidated the underlying mechanism by which CAFs promote M2 macrophage polarization and confer chemoresistance to paclitaxel, primarily through the expression of KDM2A in CAFs. Additionally, we identified the CXCR2 signaling pathway for M2 macrophage polarization and the CCL2 signaling pathway for paclitaxel resistance. Therefore, this study reveals the potential of blocking CXCR2 activation or CCL2 signaling pathway in inhibiting tumor growth and paclitaxel resistance in breast cancer.

Chemoresistance is a critical issue in cancer therapy. Chemotherapy is the standard treatment for triple-negative breast cancer (TNBC) patients in early and advanced stages. Some studies have demonstrated that upregulation of A Disintegrin and Metalloproteinase 10 (ADAM10) is associated with the chemoresistance of TNBC. Knockdown of ADAM10 decreases the IC_50_ value of paclitaxel in TNBC cells. ADAM10 promotes paclitaxel resistance via upregulating the Notch signaling pathway [38]. Additionally, previous studies have suggested that the long non-coding RNA (lncRNAs) affects the variety of genome functions and regulates the chemoresistance of breast cancer [39]. The Zeyu Xing groups have shown that LINC00337 is upregulated in breast cancer to increase the proliferation and viability of breast cancer. LINC00337 promotes the polarization of M2 macrophage to antagonize the chemotherapeutic effect of paclitaxel in breast cancer [40].

The ESTIMATE algorithm analysis relies on stromal signature genes and immune signature genes in the TCGA database and is commonly used to predict the immune and stromal state in the TME [41]. A high immune score is associated with enriched tumor-infiltrating immune cells, including CD4 + T cells, CD8 + -T cells, regulatory T (Treg) cells, and TAMs [42]. A high stromal score indicates an abundance of CAFs in the TME [43]. CD163 is a known marker expressed in M2 macrophages, while KDM2A is highly expressed in both tumor cells and CAFs. To investigate the relationship between stromal KDM2A and infiltrated M2 macrophages, we analyzed breast cancer samples with high immune/stromal scores from TCGA. This analysis revealed a positive correlation between stromal KDM2A and CD163-expressing M2 macrophages, suggesting that KDM2A-expressing CAFs are positively associated with infiltrated M2 macrophages. While the study provides compelling evidence from in vitro experiments and TCGA database analysis, further in vivo experiments are required to validate these findings.

The STAT3 inhibitor stattic majorly abolishes the phosphorylation of the STAT3 SH2 domain to inhibit the STAT3 activation; however, we found that stattic not only inhibited the STAT3 phosphorylation but also reduced the total STAT3 in IL-6 stimulated fibroblasts. Some studies suggest the same phenomenon. The Romana Mikyskova groups demonstrate that Stattic and its analogues inhibit the STAT3 phosphorylation in senescent tumor cells. The data has shown that STAT3 phosphorylation and total STAT3 expression are dose-dependently decreased in Stattic-treating proliferating tumor cells [44]. Chen Wei groups suggest that Stattic reduces the STAT3 phosphorylation and total STAT3 level in IL-6-stimulated HCT116 colorectal cancer cells [45]. The STAT3 SH2 lysine residues are also associated with STAT3 polyubiquitination and degradation [46]. Debanjan Bhattacharjee groups demonstrate that STAT3 dephosphorylation recruited the APC/CDH1 E3 ligase, resulting in STAT3 ubiquitination and degradation [47]. It has been suggested that STAT3 dephosphorylation may promote STAT3 ubiquitination. JSH-23, an inhibitor, abolishes the NFκB p65/p50 complex nuclear translocation; however, we found that total NFκB was reduced in JSH-23-treated fibroblasts. The Dandan Wu groups have seen the same phenomenon. They have demonstrated that JSH-23 reduced the NFκB p65 expression and significantly inhibited NSCLC cell proliferation, migration, and invasion [48].

## Conclusions

**Fig. 6:**
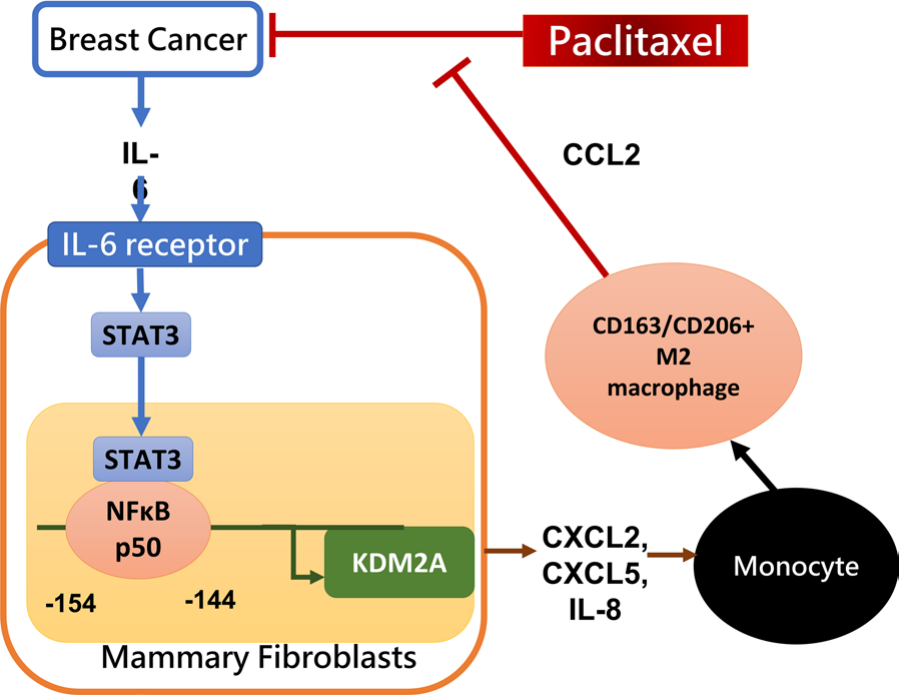
A schematic diagram of the molecular mechanism by which IL-6 secreted by breast cancer upregulates KDM2A in mammary fibroblast that can promote M2 macrophage polarization and increase paclitaxel resistance. Breast cancer cells secret IL-6, which triggers the activation of STAT3 in mammary fibroblasts. Active STAT3 then forms a complex with NFκB p50 and binds to the -154 bp to -144 bp region of the KDM2A promoter, leading to increased KDM2A expression in these fibroblasts. Elevated KDM2A levels in fibroblasts result in the release of CXCR2-assocciated chemokines, which stimulate the polarization of M2 macrophages via the CXCR2 signaling pathway. Finally, M2 macrophage release CCL2, contributing to increase resistance of breast cancer to paclitaxel. The mechanism highlights potential targets for therapeutic intervention in paclitaxel-resistant breast cancer

This study has unveiled a complex interplay within breast TME. Breast cancer-secreted IL-6 was found to orchestrate a chain of events, beginning with the upregulation of KDM2A in mammary fibroblasts through the STAT3/NFκB p50 axis. Subsequently, KDM2A-expressing CAFs played a pivotal role by releasing CXCR2-related cytokines that drove the differentiation of CD163 + /CD206 + M2 macrophages. These polarized M2 macrophages, in turn, secreted CCL2, which contributed to the heightened paclitaxel resistance in breast cancer cells. Therefore, this study highlights the potential therapeutic value of targeting the CXCR2 or CCR2 pathway as a novel strategy to combat paclitaxel-resistant breast cancer (Fig. 6).

### Supplementary Information

**Additional file 1: Figure S1**. STAT3 recruited NFκB p50 to bind to the NFκB p50 binding motif on the KDM2A promoter region and then regulated the transcription of the KDM2A gene in HS-578T CM-stimulated mammary fibroblasts. The ChIP assay was performed to pull-down STAT3 protein-chromatin complexes in HS-578T CM-incubated mammary fibroblasts using anti-STAT3 antibody. The presence of NFκB p50 binding motif on the KDM2A promoter region was amplified by PCR. Each experiment was performed in triplicate and repeated three times independently. Data are expressed as fold change relative to untreated control cells. Differences were found to be statistically significant at *p < 0.05, **p < 0.01, and ***p< 0.001.

**Additional file 2: Figure S2**. The M2 macrophage population did not significantly correlate with StromalScore. The abundance of CD163 in the tumor-dominant or TME-dominant groups was analyzed by StromalScore. There was no significant difference in the abundance of CD163 between low and high StromalScore in both the tumor-dominant and TME-dominant groups.

**Additional file 3: Figure S3**. KDM2A-expressing mammary fibroblasts did not enhance the differentiation of M1 macrophages. Expression levels of M1 macrophage marker NOS2 were analyzed in PMA-pretreated THP-1 cells co-cultured with RMF-EG cells or KDM2A-expressing RMF-EG cells (K2A-1). The relative expression of NOS2 was determined by real-time PCR assay. Differences were found to be statistically significant at *p < 0.05, **p < 0.01, and ***p < 0.001.

**Additional file 4: Figure S4**. The KDM2A-expressing fibroblast promoted M2 macrophage polarization and then increased paclitaxel resistance in MDA-MB-231 cells. Paclitaxel-treated MDA-MB-231 cells were incubated with TRMF-CM or TRK2A-CM conditioned medium for 48 hours, and the cell viability was determined. Differences were found to be statistically significant at *p < 0.05, **p <0.01, and ***p< 0.001.

**Additional file 5:  Table S1**. Primer List.

## Data Availability

The datasets generated during and/or analyzed during the current study are available from the corresponding author upon reasonable request.

## Abbreviations

KDM2A: Lysine demethylase 2A
CAFs: Cancer-associated fibroblasts
MSC: Mesenchymal stem cells
NAFs: Normal-associated fibroblasts
FAP: Fibroblast activation protein
TGFBR2: TGFβ1 receptor type 2
PTEN: Phosphatase and tensin homolog
αSMA: α-Smooth Muscle Actin
EZH2: Enhancer of zeste 2 polycomb repressive complex 2 subunit
ADAMTS1: ADAM metallopeptidase with thrombospondin type 1 motif 1
JAG-1: Jagged-1
SOX-2: SRY-box transcription factor 2
TET2: Tet-eleven translocation 2
EpCAM: Epithelial cell adhesion molecule
PD-L1: Programmed cell death-ligand 1
STAT3: Signal transducer and activator of transcription 3
NFκB: Nuclear factor-kappa B
CXCL2: C-X-C motif chemokine ligand 2
CXCL5: C-X-C motif chemokine ligand 5
CXCR2: C-X-C chemokine receptor type 2
CCL2: C–C motif chemokine ligand 2
CCR2: C–C chemokine receptor type 2
IL-6: Interleukin-6
IL-8: Interleukin-8
IL-10: Interleukin-10
VEGF: Vascular endothelial growth factor
NOS2: Nitric oxide synthase 2
TME: Tumor microenvironment
