# Supplementary figures and images for: A novel STAT3/ NFκB p50 axis regulates stromal-KDM2A to promote M2 macrophage-mediated chemoresistance in breast cancer
Source: Cancer Cell Int. 2023 Oct 11;23:237. doi: 10.1186/s12935-023-03088-1 (PMC10568766; doi:10.1186/s12935-023-03088-1)

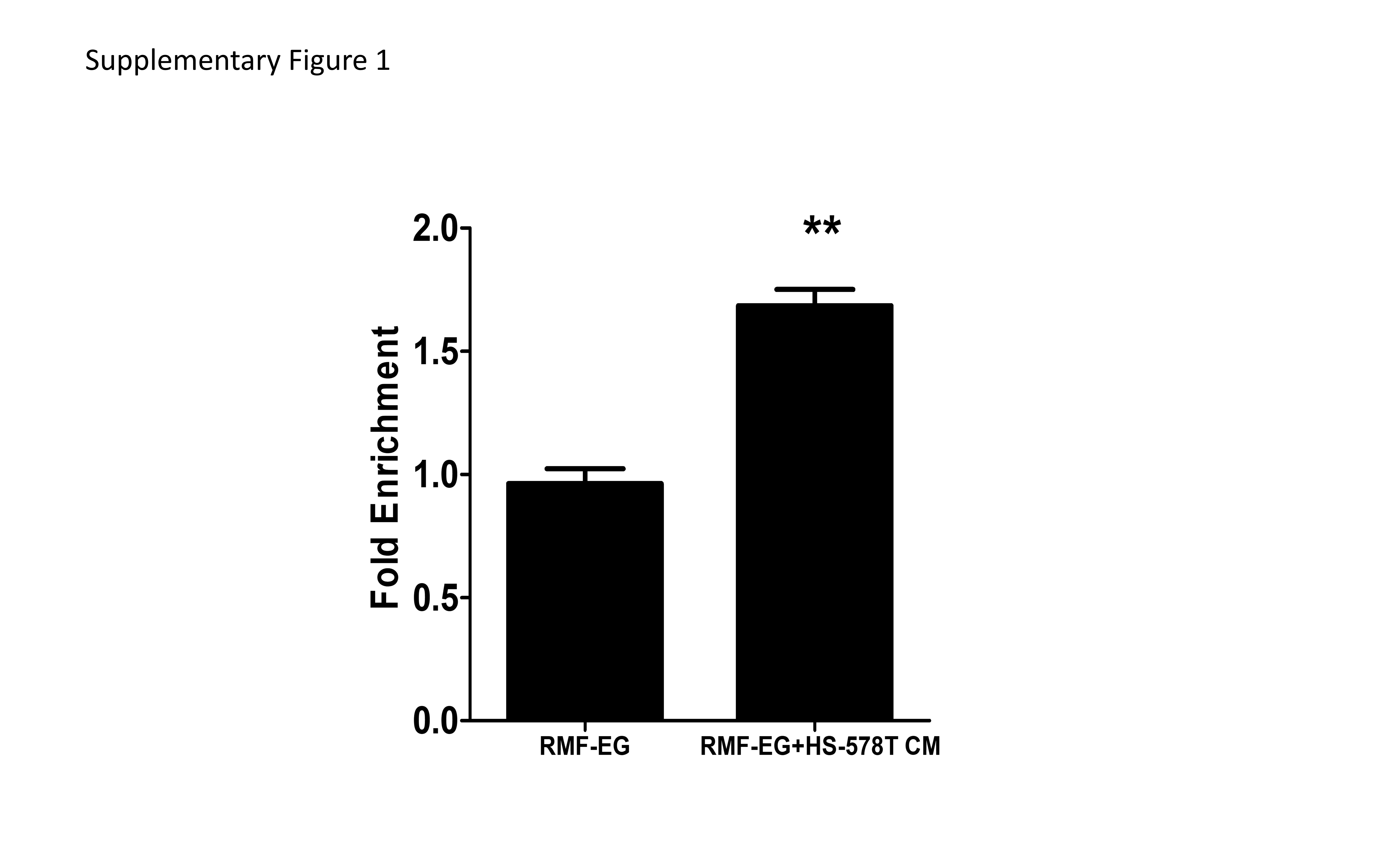

Supplement: Supplementary file 1 — Additional file 1: Figure S1. STAT3 recruited NFκB p50 to bind to the NFκB p50 binding motif on the KDM2A promoter region and then regulated the transcription of the KDM2A gene in HS-578T CM-stimulated mammary fibroblasts. The ChIP assay was performed to pull-down STAT3 protein-chromatin complexes in HS-578T CM-incubated mammary fibroblasts using anti-STAT3 antibody. The presence of NFκB p50 binding motif on the KDM2A promoter region was amplified by PCR. Each experiment was performed in triplicate and repeated three times independently. Data are expressed as fold change relative to untreated control cells. Differences were found to be statistically significant at *p < 0.05, **p < 0.01, and ***p< 0.001. [file 12935_2023_3088_MOESM1_ESM.tif]

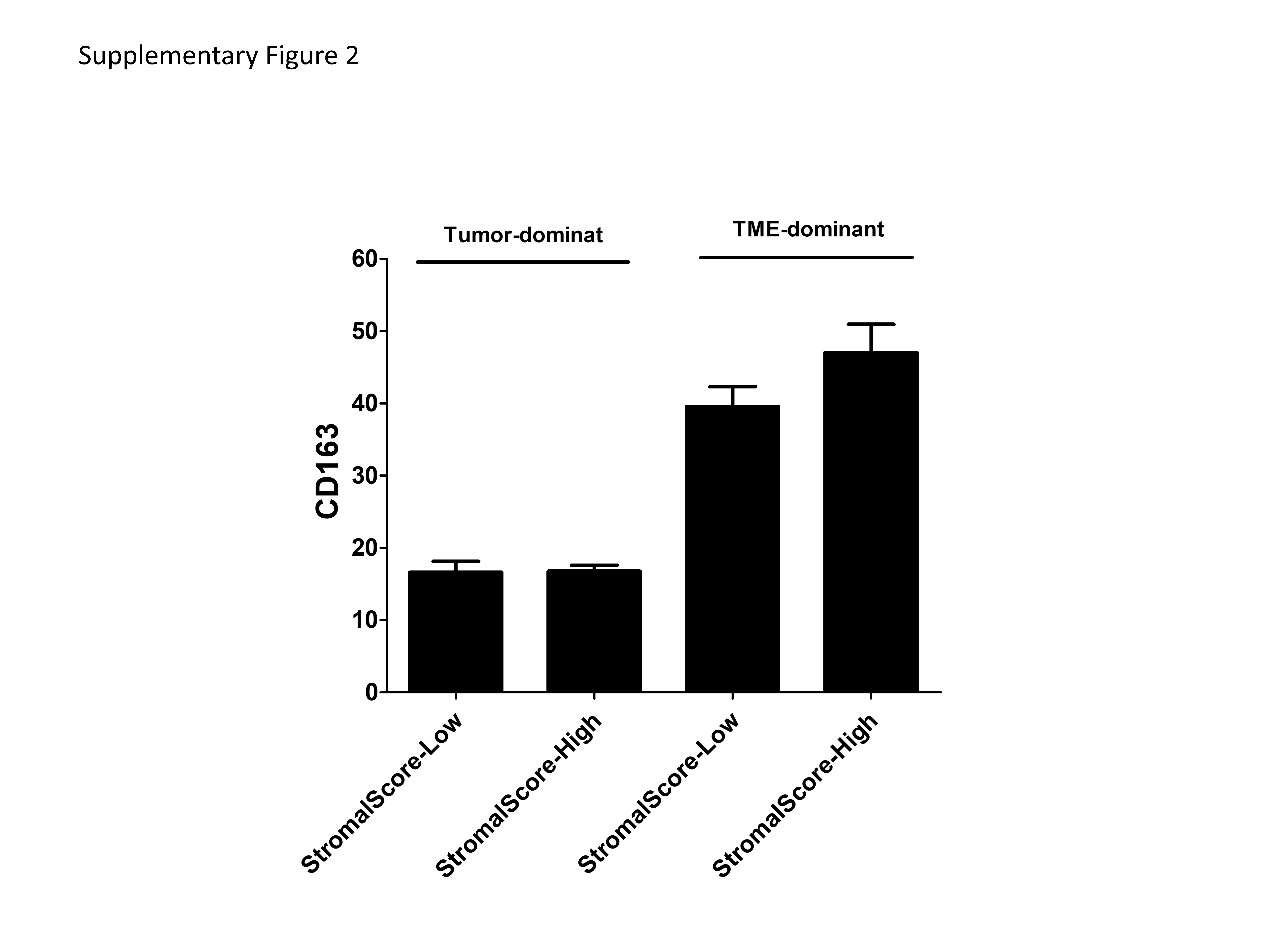

Supplement: Supplementary file 2 — Additional file 2: Figure S2. The M2 macrophage population did not significantly correlate with StromalScore. The abundance of CD163 in the tumor-dominant or TME-dominant groups was analyzed by StromalScore. There was no significant difference in the abundance of CD163 between low and high StromalScore in both the tumor-dominant and TME-dominant groups. [file 12935_2023_3088_MOESM2_ESM.tif]

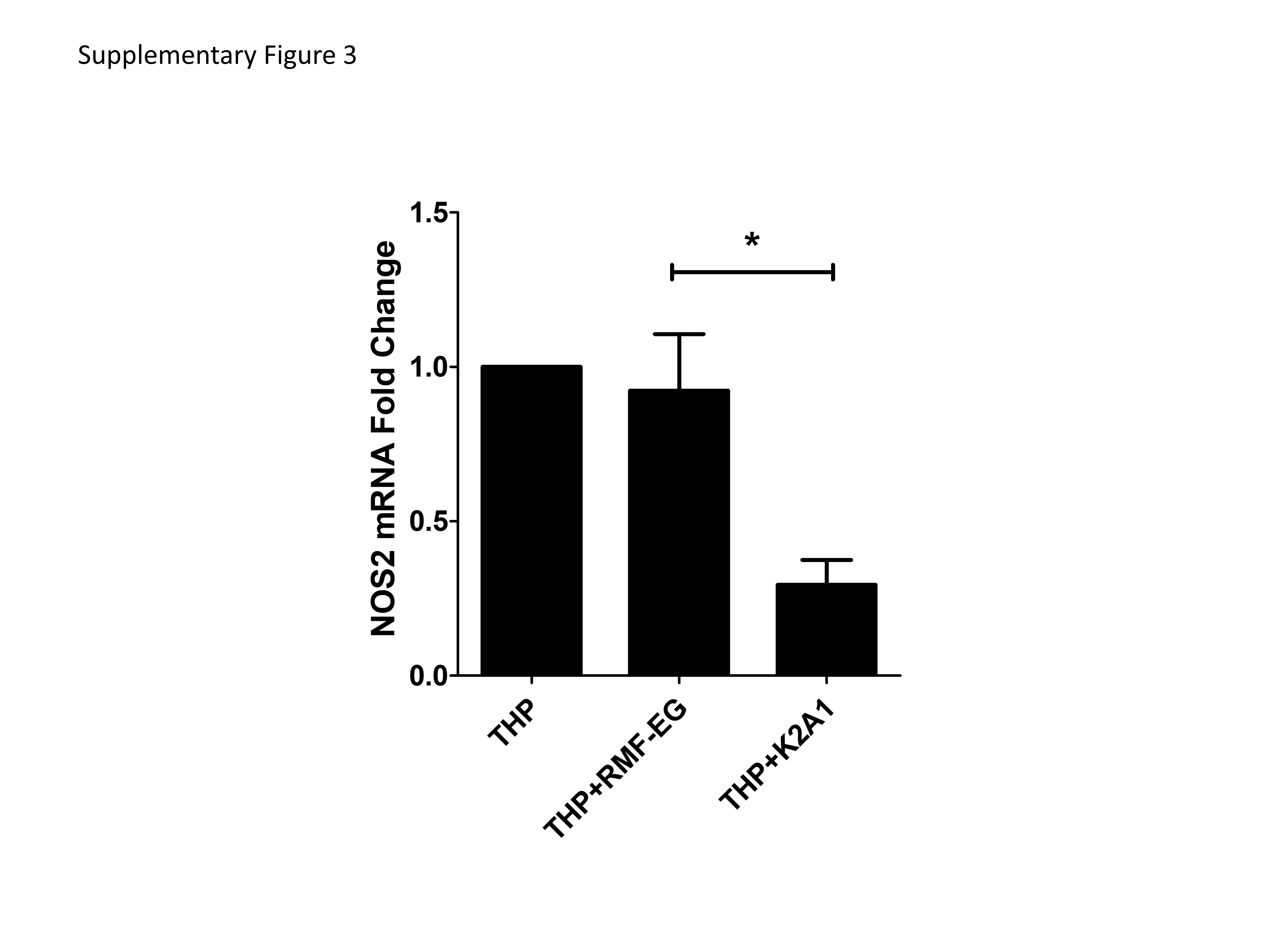

Supplement: Supplementary file 3 — Additional file 3: Figure S3. KDM2A-expressing mammary fibroblasts did not enhance the differentiation of M1 macrophages. Expression levels of M1 macrophage marker NOS2 were analyzed in PMA-pretreated THP-1 cells co-cultured with RMF-EG cells or KDM2A-expressing RMF-EG cells (K2A-1). The relative expression of NOS2 was determined by real-time PCR assay. Differences were found to be statistically significant at *p < 0.05, **p < 0.01, and ***p < 0.001. [file 12935_2023_3088_MOESM3_ESM.tif]

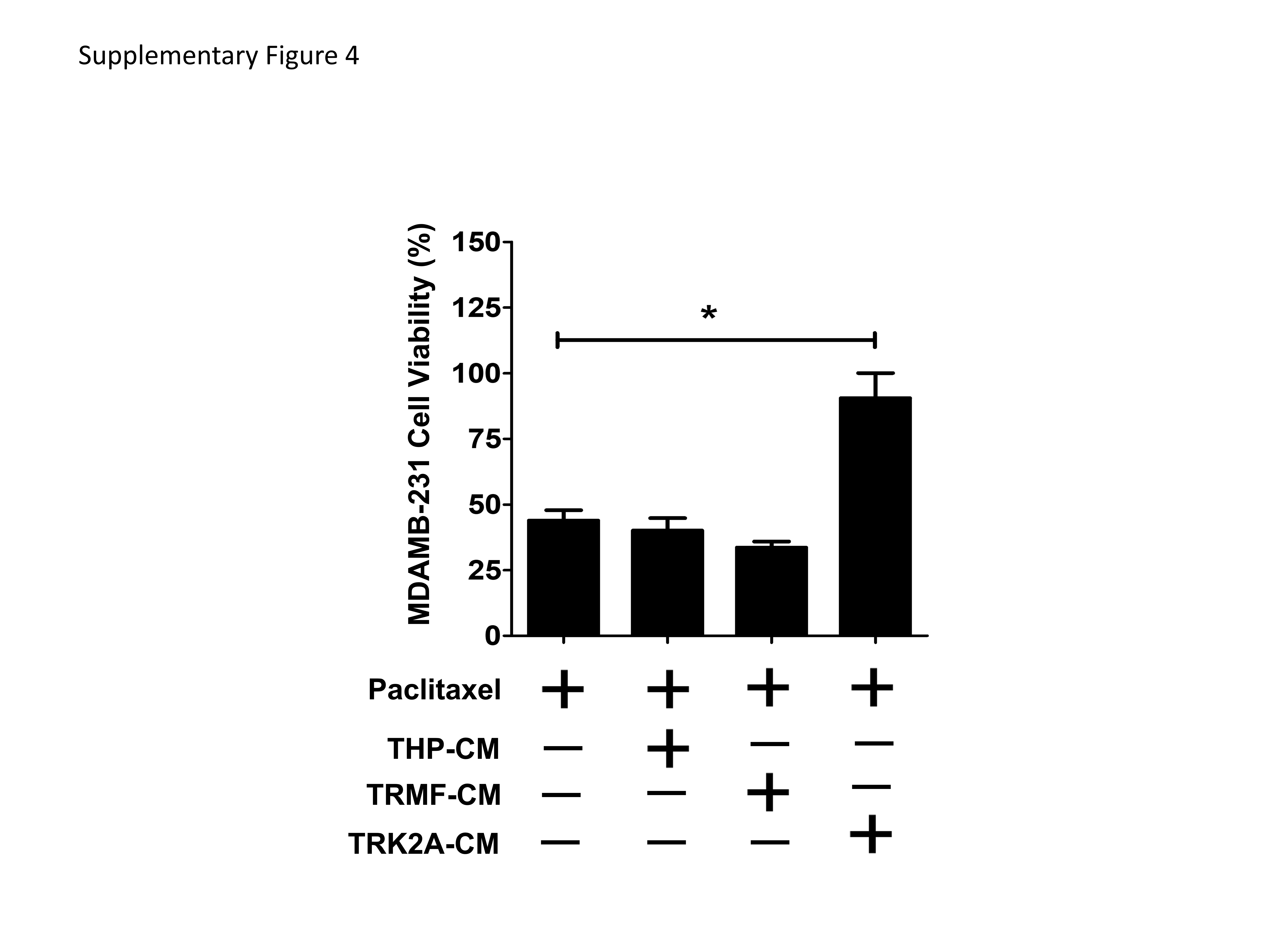

Supplement: Supplementary file 4 — Additional file 4: Figure S4. The KDM2A-expressing fibroblast promoted M2 macrophage polarization and then increased paclitaxel resistance in MDA-MB-231 cells. Paclitaxel-treated MDA-MB-231 cells were incubated with TRMF-CM or TRK2A-CM conditioned medium for 48 hours, and the cell viability was determined. Differences were found to be statistically significant at *p < 0.05, **p <0.01, and ***p< 0.001. [file 12935_2023_3088_MOESM4_ESM.tif]
