# Supplementary material for: A novel STAT3/ NFκB p50 axis regulates stromal-KDM2A to promote M2 macrophage-mediated chemoresistance in breast cancer
Source: Cancer Cell Int. 2023 Oct 11;23:237. doi: 10.1186/s12935-023-03088-1 (PMC10568766; doi:10.1186/s12935-023-03088-1)
Supplement: Supplementary file 5 — Additional file 5: Table S1. Primer List. [file 12935_2023_3088_MOESM5_ESM.docx]

**Table** S1. Primer list:

| Real-time PCR | |
| --- | --- |
| KDM2A-forward | 5’-CTCCCTTGAGCTTGGTTCTG-3’ |
| KDM2A-reverse | 5’-AATCCACTTGGGTAGCAACG-3’ |
| CCL2-forward | 5’-GATCTCAGTGCAGAGGCTCG-3 |
| CCL2-reverse | 5’-TTTGCTTGTCCAGGTGGTC-3’ |
| IL-10-forward | 5’-TTGCTGGAGGACTTTAAGGGTT-3’ |
| IL-10-reverse | 5’-TCACATGCGCCTTGATGTCT-3’ |
| VEGF-A-forward | 5’-ACAGGTACAGGGATGAGGACAC-3’ |
| VEGF-A -reverse | 5’-AAGCAGGTGAGAGTAAGCGAAG-3’ |
| CD206-forward | 5’-CAGACACGATCCGACCCTTC-3’ |
| CD206-reverse | 5’-GTCTCCGCTTCATGCCATTG-3’ |
| CD163-forward | 5’-CAGCGGCTTGCAGTTTCCTC-3’ |
| CD163-reverse | 5’-CAGCTGACTCATGGGAATTTTCTG-3’ |
| NOS2-forward | 5’-CTGGCAAGCCCAAGGTCTAT-3’ |
| NOS2-reverse | 5’-TCCCCGCAAACATAGAGGTG-3’ |
| Actin-forward | 5’-TGTTACCAACTGGGAC GACA-3’ |
| Actin-reverse | 5’-GGGGTGTTGAAGGTCTCAAA-3’ |
| Chromatin immunoprecipitation (ChIP) assay | |
| STAT4 (-319~-314) binding sites -forward | 5’-TGTGTGTGAGGGTGTGTGG-3’ |
| STAT4 (-319~-314) binding site -reverse | 5’ -CCCTCTAACGCGGACACA-3’ |
| NFκB1p50 (-154~-144) binding site-forward | 5’-CGGTGTGTCCGCGTTAGA-3’ |
| NFκB1p50 (-154~-144) binding site - reverse | 5’-CTCCTCCAGCTCGCCTCT-3’ |
